# Editorial: Perspectives and recent advances in Fetal Alcohol Spectrum Disorders research

**DOI:** 10.3389/fnins.2023.1341186

**Published:** 2023-12-15

**Authors:** Lisa K. Akison, Kirsten A. Donald, Paola A. Haeger, C. Fernando Valenzuela, Hermes H. Yeh

**Affiliations:** ^1^School of Biomedical Sciences, Faculty of Medicine, The University of Queensland, Brisbane, QLD, Australia; ^2^Department of Paediatrics and Child Health, Faculty of Health Sciences, University of Cape Town, Cape Town, South Africa; ^3^Departamento de Ciencias Biomédicas, Facultad de Medicina, Universidad Católica del Norte, Coquimbo, Chile; ^4^Department of Neurosciences, School of Medicine, University of New Mexico Health Sciences Center, Albuquerque, NM, United States; ^5^Department of Molecular and Systems Biology, Geisel School of Medicine at Dartmouth, Hanover, NH, United States

**Keywords:** fetal, alcohol, mechanisms, behavior, pathology, FASD, neurodevelopment, neuroanatomy

This *Frontiers in Neuroscience* Research Topic entitled “*Perspectives and recent advances in Fetal Alcohol Spectrum Disorders research”* was launched shortly after the 45th Annual Research Society on Alcohol Scientific Meeting held in Orlando, Florida from June 25–29, 2022. The basic concept was to provide a forum for scientific communication and cross-fertilization to advance our understanding of the short- and long-term effects and consequences of prenatal alcohol exposure (PAE) and Fetal Alcohol Spectrum Disorder (FASD). Our goal was to integrate the latest original research, reviews, methods and perspectives on foundational, translational and clinical topics in the field. In this context, we cast a wide net, inviting both preclinical and clinical articles covering diverse areas of FASD research for peer review.

FASD is the most common cause of environmentally-induced intellectual disability, and a serious public health problem. Unfortunately, our understanding of this predominantly neurodevelopmental disorder across the lifespan, as well as its etiology and mechanistic underpinnings, is incomplete. The team of Co-Editors have identified three themes that embody the twenty-eight peer reviewed articles constituting this Research Topic:

1. Effects of prenatal ethanol exposure on behavior and brain function.
2. Molecular and cellular mechanisms of ethanol-induced damage.
3. Neuroanatomical and brain structure changes due to prenatal ethanol exposure.

Collectively, the included articles highlight that FASD is a highly complex neurodevelopmental disorder with life-long consequences that calls for concerted efforts of investigation on all fronts, at all levels, and across lifespan, to advance and improve treatment and management of FASD. Although research in this field has indeed come a long way, knowledge gaps remain and must be addressed, potentially utilizing some of the advances described in this Research Topic.

## 1 Effects of prenatal ethanol exposure on behavior and brain function

The long-term impact of PAE is a subject of critical research aimed at understanding the adverse effects on brain function during adolescence and adulthood. Extensive information is available on how prenatal alcohol exposure affects various behaviors, but there is still much to discover. In this context, Chandrasekaran et al. presented new evidence regarding the influence of different types of rewards on the learning and adaptability of mice exposed to prenatal alcohol. Their study revealed that the effects of PAE vary depending on the type of reward, with liquid rewards proving to be more effective in motivating task learning in prenatally alcohol-exposed animals. Additionally, it is important to note that individuals with FASD are more likely to experience mental health issues and coping difficulties. Bodnar et al. found that while the COVID-19 pandemic did not significantly impact mental health differently between individuals with FASD and controls, it revealed distinct associations between inflammation and mood in those with FASD. This study highlights the importance of recognizing the unique challenges and heightened vulnerability of individuals with FASD during public health crises.

PAE leads to persistent neuroinflammation, suggesting this could be a potential target for future therapies. Mooney et al. demonstrated that specialized pro-resolving lipid mediator (SPM) receptors, specifically FPR2 and ChemR23 receptors involved in anti-inflammatory processes, play a significant role in anxiety and memory formation. Knockout mice with alterations in these receptors exhibit impairments, exacerbated when they are exposed to ethanol in utero. Since dietary-derived forms of omega-3 polyunsaturated fatty acids (PUFA) are ligands of these receptors, this study offers new insights into potential molecular mechanisms for dietary prevention or treatment of FASD, as well as the role of SPM receptors in mitigating cognitive impairments resulting from PAE.

Four studies highlight the influence of genetics, age, sex, and poly-drug consumption on the behavioral consequences of PAE. Baker et al. found that the effects of PAE varied significantly among mouse strains, with some strains showing minimal effects and others displaying substantial changes in hippocampal-dependent behavior. They also noted strain-sex interactions underscoring the complex interplay that determines behavioral outcomes. However, sexual dimorphism was dependent on the type of behavior analyzed. Furthermore, Bariselli et al. observed in a mouse model of moderate postnatal ethanol exposure (third-trimester equivalent) that female, but not male, mice exhibited mild social impairments and altered extinction of operant responding. However, Wang et al. discovered that heavy PAE mimicking second-trimester exposure in humans resulted in persistent anxiety-like behavior in both male and female rats. This suggests that the timing and dose of exposure is important. Interestingly, acute stress was found to mask the effects of PAE in males but not females. Additionally, Lei et al. investigated the combined effects of PAE and Δ-9-tetrahydrocannabinol on cognitive and emotional development, revealing substance- and sex-specific patterns of impairment and highlighting the need for public health policies addressing the use of alcohol and cannabis during pregnancy. The findings of these studies underscore the enduring impact of PAE on mental health and emphasize the significance of considering sex and co-exposures in understanding the risk for development of FASD.

FASD prevalence is often underestimated due to limited diagnostic tools and the absence of self-reporting of alcohol consumption. In this context, Panton et al. evaluated a multi-site project to improve FASD diagnosis and awareness in Australia. The project's success in refining FASD diagnosis and enhancing community education illustrates the importance of coordinated efforts to address the challenges of FASD diagnosis and management.

A final article in this theme sheds light on a critical but understudied aspect of FASD—the aggression displayed by affected children and youth toward their family members. The review by Champagne et al. emphasizes the lack of interventions targeting this specific behavior and underscores the urgent need for research in this area.

Taken together, these clinical and preclinical studies highlight the complex factors underlying the risk for the development of behaviors characteristic of FASD, as well as the challenges in diagnosis and management of this condition.

## 2 Molecular and cellular mechanisms of ethanol-induced damage

Several studies in this theme contribute significant insights into the effects of ethanol on transcription and translation in the developing brain. Holloway et al. focused on early transcriptomic changes in the cerebellum in a mouse model of FASD, identifying key pathways and cellular functions altered by PAE, including immune function, cytokine signaling, and cell cycle regulation. Notably, there was an increase in transcripts associated with neurodegenerative microglia and reactive astrocyte phenotypes. Hashimoto et al. characterized the impact of ethanol exposure on astrocytes using translating ribosome affinity purification (TRAP). They found significant overlap between ethanol-affected genes in the total RNA pool and the translating RNA pool. Moreover, the overlap between ethanol-regulated genes in this study and other *in vivo* exposure models supports the generalizability of these findings to various PAE scenarios. Ruffaner-Hanson et al. studied the impact of PAE on stress-responsive brain regions in mice, emphasizing sex-dependent responses. PAE resulted in dysregulated neuroendocrine and neuroimmune activation, primarily in females. The findings highlight the importance of considering sex-specific effects and the role of Toll-like receptor 4 (TLR4) signaling in the context of PAE. The study by Papageorgiou et al. is the first to highlight the emerging role of circular RNAs (circRNAs) in the context of PAE, demonstrating sex- and brain region-specific alterations in circRNAs as well as in long non-coding RNA (lncRNA) expression. Notably, CircHomer1, a circRNA abundant in the postnatal brain, is significantly reduced in the frontal cortex and hippocampus of male mice exposed to moderate PAE. Meanwhile, there was an increase in the expression of the embryonic brain-enriched lncRNA H19 in the frontal cortex of these male PAE mice. In a related study by Noor et al., PAE led to alterations in the levels of circRNAs associated with immune function and suggested a potential link between PAE-induced neuroimmune actions and adult-onset pain dysregulation. Lastly, Davies et al. examined the effects of PAE on gene expression of two histamine H3 receptor isoforms (rH_3A_ and rH_3C_) in different brain regions in the rat. Interestingly, changes were detected in brain regions where the investigators had previously observed PAE-induced changes in H3R-effector coupling.

Two studies specifically examined the role of mitochondria and oxidative stress in the mechanism of action of PAE. Mazumdar and Eberhart addressed the genetic factors influencing susceptibility to ethanol-induced birth defects, focusing on the role of nicotinamide nucleotide transhydrogenase (Nnt) in mitigating oxidative stress. This study, conducted in zebrafish embryos, demonstrated that ethanol exposure leads to increased apoptosis and craniofacial malformations in Nnt mutants due to elevated reactive oxygen species. Importantly, antioxidant treatment rescued these defects. To further explore the mechanisms underlying PAE-induced oxidative stress, Darbinian et al. examined mitochondrial DNA (mtDNA) in fetal brain tissues from PAE-exposed rats and humans, as well as fetal brain-derived exosomes (FB-Es) obtained from maternal blood. Human tissues of this type with complementary records of maternal alcohol exposure are rarely available. PAE resulted in mtDNA damage in both the rat and human fetal brain tissue, as well as FB-Es from maternal blood. mtDNA damage in FB-Es also correlated with reduced fetal eye size. This suggests that FB-Es may be useful clinically as a surrogate marker for mtDNA damage in the fetal brain and thus predict risk for FASD. *In vitro* treatment of neuronal cells with ethanol also showed mtDNA damage leading to oxidative stress and apoptosis while insulin-like growth factor 1 (IGF1) was shown to rescue mtDNA from this damage.

Two studies delved into potential factors that may modulate the effect of PAE. Upreti et al. introduced the gut microbiota as a potential factor in FASD pathophysiology. The link between gut microbiota and neurodevelopmental disorders was explored, suggesting that microbiota dysbiosis may play a role in the development of FASD. This area of research is in its early stages but holds promise in understanding the long-term health effects of PAE. Choline has been identified as a potential supplement to ameliorate behavioral, neurological, and cognitive deficits from PAE (see Akison et al., 2018 for a review of preclinical and Ernst et al., 2022 for a review of clinical studies). Xu et al. investigated the potential therapeutic benefits of choline in mitigating ethanol-induced cell death in the developing neural tube using BXD strains of mice, known to vary in their sensitivity to ethanol's teratogenic effects (Downing et al., 2012). Choline administration effectively reduced cell death in all strains, without causing harm in un-exposed mice. However, there were some dose-dependent differences across strains and brain regions, indicating that there is genetic variability in the response to choline treatment as well as ethanol sensitivity.

A final study in this theme investigated the role of somatostatin (SST) GABAergic neurons in sleep circuitry and their sensitivity to developmental ethanol exposure (Wilson et al.). Optical activation of prefrontal cortex SST neurons induced slow-wave potentials and delayed single-unit excitation in mice treated with saline but not in those exposed to ethanol during the brain growth spurt (third-trimester equivalent). When these neurons were activated in a closed-loop manner during spontaneous slow waves, it enhanced cortical delta oscillations, an effect that was more pronounced in saline-treated mice compared to ethanol-exposed mice. These findings indicate that SST cortical neurons may play a role in the impaired slow-wave activity observed after PAE.

These studies collectively show the multifaceted molecular and cellular mechanisms underlying PAE-induced damage in the brain. These include early transcriptomic changes, genetic factors, mitochondrial dysfunction, stress responses, gut microbiota dysbiosis, and the role of specific molecular players like circRNAs, lncRNAs, somatostatin neurons, and histamine receptors. Moreover, these investigations highlight the significance of considering sex-specific effects and genetic variability in response to interventions.

## 3 Neuroanatomical and brain structure changes due to prenatal ethanol exposure

While FASD has recently been recognized as a ‘whole body diagnosis' (Himmelreich et al., 2020), undoubtedly the major focus of research is on the impacts of the teratogenic effects of ethanol on the developing brain. However, the available diagnostic criteria for FASD do not include specific neuroanatomical or structural brain changes. There are well-documented reductions in brain size, but there is less consistency in reports of specific anatomical markers that may provide a reliable phenotype for prenatal alcohol-induced damage. Studies investigating the complex relationship between PAE, structural brain changes, and cognitive and behavioral outcomes, highlight the need for prevention strategies as well as tailored interventions in children with prenatal exposure to alcohol.

Four clinical studies used innovative methods and approaches to explore morphological changes in the brain induced by PAE that may be potentially responsible for FASD-related cognitive and behavioral deficits. Boateng et al. used structural magnetic resonance imaging (MRI) to measure overall brain volume, as well as volume and surface area of specific brain regions, in children diagnosed with FASD and PAE compared to typically developing controls. While confirming previous reports of smaller brain volume in children with FASD/PAE (e.g., Treit et al., 2016), only specific FASD subtypes (pFAS/FAS) showed reduced volume in specific regions of the brain, notably the cerebellum, caudate, and pallidum. Also, for those tested, there was a significant correlation between volume and IQ.

Although focal abnormalities in the corpus callosum (CC) have been frequently reported clinically at various ages (see Moore and Xia, 2022 for review), Fraize et al. used an innovative method combining sulci-based cortical segmentation and connectivity-based parcellation of the CC to explore this further. They found that various CC parcels and corresponding cortical regions were smaller in individuals with FASD compared to controls. For example, even when adjusting for confounding factors such as age, sex and brain size, the postcentral callosal parcel and its corresponding cortical region appeared to be persistently affected. Given the importance of the CC in interhemispheric communication in the brain, it is perhaps not surprising that the reduced size of these CC parcels may provide a clinically relevant neuroanatomical marker for FASD diagnosis and at least a partial explanation for the cognitive and motor deficits in individuals with FASD (Biffen et al., 2022). Additionally, Gimbel et al. used a combination of MRI tractography with a novel diffusion-weighted Neurite Orientation Dispersion and Density Imaging (NODDI) model to characterize the impact of PAE on longitudinal trajectories of developmental white matter microstructure. PAE resulted in atypical developmental trajectories from childhood (~9 years) through adolescence (~17 years). There were also sex-specific differences detected, particularly in the neurite density index, with males with PAE more severely affected than females.

Finally, Ostertag et al. used diffusion tensor imaging (DTI) to examine the arcuate fasciculus (AF), a white matter tract primarily in the left hemisphere of the brain that is important for language processing. Children with FASD are reported to have deficits in articulation, grammatical ability and both expressive and receptive language (Mattson et al., 2019) and therefore developmental impairment of the AF may help to explain these deficits. The results indicated that children with PAE show altered developmental trajectories of the AF, which were associated with lower pre-reading language ability, including phonological processing and speeded naming, well described as being affected in this group.

Three preclinical studies delved deeper into the cellular level effects of ethanol exposure during brain development, and investigated a potential therapeutic. Cealie et al. used a mouse-model of human third-trimester exposure to specifically focus on microglia dynamics, morphology and interactions with Purkinje cells within the cerebellum during offspring adolescence. Microglia are considered the ‘immune cells of the brain' (Eyo and Wu, 2019) and have been shown to be susceptible to ethanol-induced cell death and altered phenotype in surviving cells (Kane et al., 2011). Perhaps surprisingly, PAE at this time-point produced minimal long-term effects on cerebellar microglia and their interactions with neurons, suggesting that at least in this region of the brain, these cells are relatively resilient when exposed to alcohol during this phase of development. However, a mouse study estimating neuron numbers in cortical and sub-cortical regions following PAE at a similar time-point (third trimester equivalent) paints a different picture. Smiley et al. used stereological cell counting to compare immediate (8h post) and long-term (post-natal day 70) neuron loss and showed both an immediate and lasting reduction in neuron numbers in various brain regions. This highlights the profound and persistent impact of neonatal ethanol exposure on neuron numbers in the developing brain. The study by Saito et al. explores the potential therapeutic effects of retinoic acid receptor α (RARα) modulators on ethanol-induced neurodegeneration and neuroinflammation in neonatal mice. The findings suggest that RARα antagonist partially blocks acute neurodegeneration and phagocytic cell elevation. On the other hand, an RARα agonist ameliorates long-lasting astrocyte activation and GABAergic cell deficits. These results indicate that modulating RARα signaling may have potential therapeutic benefits in mitigating the neurological consequences of developmental ethanol exposure. The study highlights the complex interplay between retinoic acid signaling, neurodegeneration, and neuroinflammation in FASD, offering insights into potential avenues for treatment.

Although not specifically focused on brain structural changes, Blanck-Lubarsch et al. provided a comprehensive systematic review of orofacial structural abnormalities in patients with FAS specifically, as well as across the FASD diagnostic spectrum. Dysmorphology in several orofacial features are typically part of the diagnostic criteria for FAS, but they are not used consistently across the various guidelines currently available. Additionally, there are other dysmorphologies which may prove to be potential diagnostic criterion across the spectrum. The review included 61 clinical studies evaluating various facial, oral, dental, and orthodontic features in patients with FASD. The most common orofacial features measured included palpebral fissure length, interpupillary distance, philtrum, upper lip, midfacial hypoplasia, and head circumference. The review emphasized the need for standardized and objective diagnostic criteria for orofacial features in FASD. Such criteria would enhance diagnostic accuracy and consistency, facilitating early identification and intervention for affected individuals.

Overall, while the preclinical studies advance our understanding of neurobiological mechanisms underpinning the developmental effects of PAE, the multimodal range of neuroimaging in clinical populations improves our understanding of potential neuro-physical features of FASD. Taken together, these studies provide an exploration of trajectories across the important early adolescent developmental window and potential targets for diagnosis and therapeutic interventions.

## Author contributions

LA: Writing—original draft, Writing—review & editing. PH: Writing—original draft, Writing—review & editing. KD: Writing—original draft, Writing—review & editing. CV: Conceptualization, Writing—original draft, Writing—review & editing. HY: Conceptualization, Writing—original draft, Writing—review & editing.
